# PD-L1 immunohistochemistry for canine cancers and clinical benefit of anti-PD-L1 antibody in dogs with pulmonary metastatic oral malignant melanoma

**DOI:** 10.1038/s41698-021-00147-6

**Published:** 2021-02-12

**Authors:** Naoya Maekawa, Satoru Konnai, Maki Nishimura, Yumiko Kagawa, Satoshi Takagi, Kenji Hosoya, Hiroshi Ohta, Sangho Kim, Tomohiro Okagawa, Yusuke Izumi, Tatsuya Deguchi, Yukinari Kato, Satoshi Yamamoto, Keiichi Yamamoto, Mikihiro Toda, Chie Nakajima, Yasuhiko Suzuki, Shiro Murata, Kazuhiko Ohashi

**Affiliations:** 1 Department of Advanced Pharmaceutics, Faculty of Veterinary Medicine, Hokkaido University, Sapporo, Japan; 2 Department of Disease Control, Faculty of Veterinary Medicine, Hokkaido University, Sapporo, Japan; 3 North Lab, Sapporo, Japan; 4 Veterinary Teaching Hospital, Faculty of Veterinary Medicine, Hokkaido University, Sapporo, Japan; 5 Department of Veterinary Surgery 1, School of Veterinary Medicine, Azabu University, Sagamihara, Japan; 6 Department of Antibody Drug Development, Tohoku University Graduate School of Medicine, Sendai, Japan; 7 New Industry Creation Hatchery Center, Tohoku University, Sendai, Japan; 8 Fuso Pharmaceutical Industries, Ltd., Osaka, Japan; 9 Research Center for Zoonosis Control, Hokkaido University, Sapporo, Japan; 10 Global Station for Zoonosis Control, Global Institution for Collaborative Research and Education (GI-CoRE), Hokkaido University, Sapporo, Japan

**Keywords:** Immunotherapy, Cancer immunotherapy

## Abstract

Immunotherapy targeting programmed cell death 1 (PD-1) and PD-ligand 1 (PD-L1) represents promising treatments for human cancers. Our previous studies demonstrated PD-L1 overexpression in some canine cancers, and suggested the therapeutic potential of a canine chimeric anti-PD-L1 monoclonal antibody (c4G12). However, such evidence is scarce, limiting the clinical application in dogs. In the present report, canine PD-L1 expression was assessed in various cancer types, using a new anti-PD-L1 mAb, 6C11-3A11, and the safety and efficacy of c4G12 were explored in 29 dogs with pulmonary metastatic oral malignant melanoma (OMM). PD-L1 expression was detected in most canine malignant cancers including OMM, and survival was significantly longer in the c4G12 treatment group (median 143 days) when compared to a historical control group (*n* = 15, median 54 days). In dogs with measurable disease (*n* = 13), one dog (7.7%) experienced a complete response. Treatment-related adverse events of any grade were observed in 15 dogs (51.7%). Here we show that PD-L1 is a promising target for cancer immunotherapy in dogs, and dogs could be a useful large animal model for human cancer research.

## Introduction

Therapeutic antibodies targeting immune checkpoint molecules, such as programmed cell death 1 (PD-1) and its ligand PD-ligand 1 (PD-L1), have shown great promise in cancer treatment, by reinvigorating immune responses against cancers^1^. PD-1 is an immunoinhibitory receptor which belongs to the CD28 family, and expressed predominantly in activated T cells^2^. PD-L1 is expressed in various cell types, including both hematopoietic and nonhematopoietic cells^2^. The interaction of PD-1 with PD-L1 attenuates T-cell effector functions, including cytokine secretion and cell proliferation, that are essential for robust immune responses^3^. Of note, PD-L1 overexpression is often reported in cancers, suggesting the PD-1/PD-L1 axis is a possible mechanism adopted by cancers for immune evasion^4,5^. Blockade of these molecules using monoclonal antibodies (mAbs) enhances T-cell responses against cancer antigens in both mouse models and in vitro human studies^6–8^.

Nivolumab, an anti-PD-1 mAb, demonstrated safety and efficacy in patients with advanced melanoma, non-small-cell lung cancer (NSCLC), and renal cell carcinoma. However, while the authors reported treatment response in 36% of patients with PD-L1-positive cancers, no objective response was found in PD-L1–negative cancer patients^9^. In another study, NSCLC patients treated with pembrolizumab showed objective response rate (ORR) of 45.2% if ≥50% of the tumor cells were PD-L1-positive on immunohistochemistry (IHC), whereas only 16.5% of patients responded to treatment if 1–49% of the tumor cells were PD-L1-positive^10^. These studies suggest PD-L1 expression in cancers may represent a useful biomarker for clinical response to PD-1/PD-L1-inhibiting mAbs. To date, although PD-L1 IHC alone is not considered a precise biomarker^11^, the development of sensitive PD-L1 IHC could provide a rationale for the use of PD-1/PD-L1-inhibiting mAbs.

Malignant melanoma is a relatively common, but fatal disease in dogs, especially with distant metastasis (stage IV: World Health Organization staging^12^). Among canine malignant melanoma, oral malignant melanoma (OMM) is characterized by high invasiveness and metastatic propensity^13,14^. The median survival of dogs with stage IV OMM has been reported as 80 days, when treated with radiation^15^. The lungs are common sites for distant metastasis, and dogs with pulmonary metastatic OMM have the median survival of less than 2 months, when treated with existing therapies including surgery, radiation, and/or chemotherapy^16^. Metastatic lesions appear resistant to chemotherapy^17^, and the lack of effective systemic therapy leads to very short expected survival. Therefore, the development of new systemic therapy is crucial.

Recent studies have demonstrated that the PD-1/PD-L1 pathway is also involved in immune evasion of canine cancers. Some canine cancers, including OMM, were reported to express PD-L1, and specific anti-PD-1/PD-L1 mAbs induced immune-cell activation in vitro^18–21^. However, few studies have attempted to assess PD-L1 expression in various canine cancers, and there is currently no consensus on PD-L1 expression status in each cancer type. We therefore aimed to assess PD-L1 expression in various cancer types, including OMM, using a novel anti-PD-L1 mAb (6C11-3A11) and compared its sensitivity to 6G7-E1, a previously reported mAb for canine IHC^19^.

In addition, a recent pilot study demonstrated antitumor efficacy of a canine chimeric anti-PD-L1 mAb (c4G12) against canine OMM and undifferentiated sarcoma (*n* = 2), among a sample of 9 dogs^16^. We therefore further aimed to assess the efficacy and safety of c4G12, in dogs with pulmonary metastatic OMM, using a predetermined dosage regimen^16^. A secondary objective included the exploration of factors predictive of survival, including known correlates of improved survival in anti-PD-1/PD-L1 therapy-treated patients; use of radiation therapy, C reactive protein (CRP) level and lymphocyte-to-monocyte ratio (LMR)^22–24^.

## Results

### PD-L1 IHC using 6C11-3A11 in various canine malignant cancers

We previously established a canine PD-L1 IHC using 6G7-E1, an IgM class mAb^19^. However, the sensitivity of our IHC seemed insufficient due to a lack of demonstrated PD-L1 expression in several cancer types, contrary to other reports^19,20,25,26^. To resolve this inconsistency, we obtained a mAb from our hybridoma pool, named 6C11-3A11^27,28^. In human, normal tonsil is used as positive and negative control for PD-L1 IHC^29^. In 6C11-3A11 IHC, canine tonsil epithelium was stained positively (Supplementary Fig. 1), while squamous epithelium in the same section showed no specific signals. The staining pattern appeared to be consistent with that in human tonsils, suggesting the specificity of 6C11-3A11 IHC. Next, we performed IHC with both 6C11-3A11 and 6G7-E1 and directly compared the staining intensity and patterns using the same cancer samples. In squamous cell carcinoma, nasal adenocarcinoma, transitional cell carcinoma, anal sac gland carcinoma, and soft tissue sarcoma, specific staining was not observed using 6G7-E1. However, most of these samples showed clear positive signals in 6C11-3A11 IHC. In osteosarcoma samples, specific staining was confirmed by both mAbs, and expectedly, 6C11-3A11 produced higher staining intensity (*n* = 5 for each cancer type, Supplementary Fig. 2 and Supplementary Table 1). Taken together, this suggests that 6C11-3A11 is more sensitive than 6G7-E1 for the detection of PD-L1 in IHC.

Next, PD-L1 expression was further investigated in various malignant cancers by 6C11-3A11 IHC. Among the 20 samples tested, 18 (90%), 20 (100%), 20 (100%), 19 (95%), 14 (70%), and 20 (100%) were PD-L1-positive for squamous cell carcinoma, nasal adenocarcinoma, transitional cell carcinoma, anal sac gland carcinoma, soft tissue sarcoma, and osteosarcoma, respectively. In 19 (95%) of OMM, 20 (100%) of mammary adenocarcinoma, 18 (90%) of histiocytic sarcoma, 17 (85%) of diffuse large B-cell lymphoma, and 4 (80%) of gastric adenocarcinoma, PD-L1 was detected in the tumor cells (Supplementary Fig. 3a–e). In contrast, none of the transmissible venereal tumor specimens expressed PD-L1 (Supplementary Fig. 3f). In most cases, tumor cells were stained intracellularly, whereas cell surface staining was obscure. Of note, infiltrating plasma cells and lymphocytes were also PD-L1-positive in some specimens, while stromal cells surrounding the tumor cells were predominantly PD-L1-negative.

**Fig. 1:**
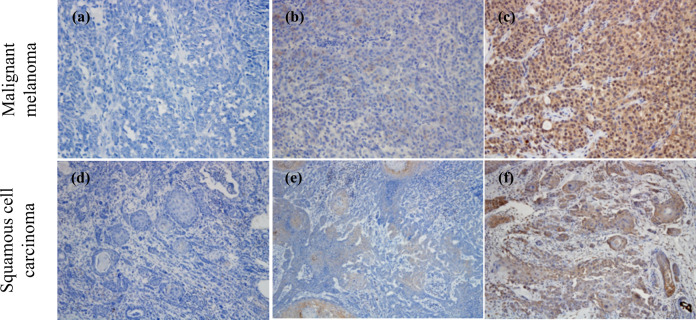
Representative PD-L1 immunohistochemistry results with 6C11-3A11 for each tumor proportion score (TPS). The proportion of PD-L1–expressing tumor cells was scored according to the percent of stained viable tumor cells (see “Methods”). Representative IHC results for TPS < 1% (**a**, **d**), 1–49% (**b**, **e**), and ≥50% (**c**, **f**) are shown. Original magnification, ×200 (**a**–**c**) or ×100 (**d**–**f**).

To develop a scoring protocol for canine PD-L1 IHC, tumor proportion score (TPS) for PD-L1 expression was calculated according to the criteria described in the Methods section. The majority of samples from each cancer type had TPS of ≥50%, except for histiocytic sarcoma, in which most of the samples had TPS of 1–49%. Table 1 summarizes the results of PD-L1 IHC with 6C11-3A11. Representative IHC results for malignant melanoma and squamous cell carcinoma are shown in Fig. 1, with examples for each TPS.

**Table 1:** PD-L1 expression in various malignant cancers.

Pathology	Positive no./tested no.	Positive rate (%)	PD-L1 TPS		
			<1%	1–49%	≥50%
Squamous cell carcinoma (Skin)	18/20	90	2	2	16
Nasal adenocarcinoma	20/20	100	0	2	18
Transitional cell carcinoma	20/20	100	0	0	20
Anal sac gland carcinoma	19/20	95	1	4	15
Soft tissue sarcoma	14/20	70	6	2	12
Osteosarcoma	20/20	100	0	4	16
Malignant melanoma (Oral)	19/20	95	1	1	18
Mammary adenocarcinoma	20/20	100	0	0	20
Histiocytic sarcoma	18/20	90	2	12	6
Diffuse large B-cell lymphoma	17/20	85	3	5	12
Gastric adenocarcinoma	4/5	80	1	1	3
Transmissible venereal tumor	0/4	0	4	0	0

*TPS* tumor proportion score

### Characteristics of dogs enrolled in clinical study using c4G12

To evaluate the safety and clinical benefits of anti-PD-L1 mAb in canine pulmonary metastatic OMM, we conducted a veterinary clinical study of c4G12 in our hospital involving 29 dogs (see Supplementary Table 2 for details of each dog). At the time of study enrollment, the median age was 13 years (range: 8–16 years). All dogs had primary OMM diagnosed by histopathological assessment and pulmonary metastases (PM) were confirmed by chest X-ray or computed tomography (CT) scan. Prior to the enrollment, most dogs underwent at least one prior treatment, including surgery, radiation, and/or chemotherapy. The majority of dogs had PD-L1-positive cancers with TPS of ≥50%, whereas only 2 dogs had PD-L1–negative cancers (TPS < 1%). At baseline, 13 dogs (44.8%) had measurable disease as defined by cRECIST v1.0^30^. The baseline characteristics of dogs are summarized in Table 2.

**Table 2:** Characteristics of the dogs at baseline.

Characteristic	
Age—year	
Median	13
Range	8–16
Age category―no. (%)	
≥10 year	27 (93.1)
<10 year	2 (6.9)
Sex―no. (%)	
Male	
Intact	8 (27.6)
Castrated	11 (37.9)
All	19 (65.5)
Female	
Intact	3 (10.3)
Spayed	7 (24.1)
All	10 (34.5)
PD-L1 status—no. (%)	
Positive	
TPS ≥ 50%	18 (62.1)
TPS 1–49%	1 (3.4)
TPS ND	7 (24.1)
All	26 (89.7)
Negative (TPS < 1%)	2 (6.9)
ND	1 (3.4)
Prior therapy―no. (%)	
Surgery	17 (58.6)
Radiation	
≤8 weeks^a^	8 (27.6)
>8 weeks	4 (13.8)
Unknown	2 (6.9)
All	14 (48.3)
Chemotherapy	1 (3.4)
None	2 (6.9)
Measurable lesion(s)―no. (%)	
Present	13 (44.8)
Absent	16 (55.2)

*TPS* tumor proportion score, *ND* not determined.

^a^Dogs that received previous radiation ≤8 weeks before the first dose of c4G12.

### Safety of c4G12 treatment

Dogs were treated with intravenous administration of c4G12 every 2 weeks. Median duration of c4G12 treatment was 98 days (range: 15–518 days). Concomitant therapy including radiation and surgical excision was allowed in order to achieve local tumor control (see Supplementary Table 3 for details of the treatment of each dog). Treatment-related adverse events (TRAEs) of any grade were observed in 15–29 dogs (51.7%). TRAEs that occurred in at least 10% of dogs included vomiting, diarrhea, and elevated ALT, AST, and Lipase. Grade 3 TRAEs were observed in 4 dogs (13.8%), including elevated ALT, AST, and Lipase without any clinical symptoms. One dog developed grade 3 pneumonitis after the second dose of c4G12, but recovered with treatment discontinuation and supportive care including glucocorticoid administration. No grade 4 or 5 TRAEs were observed throughout the study. All TRAEs are listed in Table 3.

**Table 3:** Treatment-related adverse events (TRAEs).

TRAEs—no. (%)	Any grade	Grade 3	Leading to discontinuation
Any TRAEs	15 (51.7)	4 (13.8)	1 (3.4)
Anorexia	1 (3.4)	0 (0)	0 (0)
Vomiting	4 (13.8)	0 (0)	0 (0)
Diarrhoea	3 (10.8)	0 (0)	0 (0)
Thrombocytopenia	2 (6.9)	0 (0)	0 (0)
Albumin, low	1 (3.4)	0 (0)	0 (0)
ALT	8 (27.6)	1 (3.4)	0 (0)
AST	3 (10.3)	1 (3.4)	0 (0)
Alkaline phosphatase	1 (3.4)	0 (0)	0 (0)
Lipase	3 (10.3)	1 (3.4)	0 (0)
CPK	1 (3.4)	0 (0)	0 (0)
Conjunctivitis/ocular surface disease	1 (3.4)	0 (0)	0 (0)
Pneumonitis/pulmonary infiltrates	1 (3.4)	1 (3.4)	1 (3.4)

Adverse events that were considered to be related to the treatment are listed. Grading was performed according to VCOG-CTCAE v1.1^54^.

*ALT* alanine aminotransferase, *AST* aspartate aminotransferase, *CPK* creatine phosphokinase.

### Clinical efficacy of c4G12 treatment

**Fig. 2:**
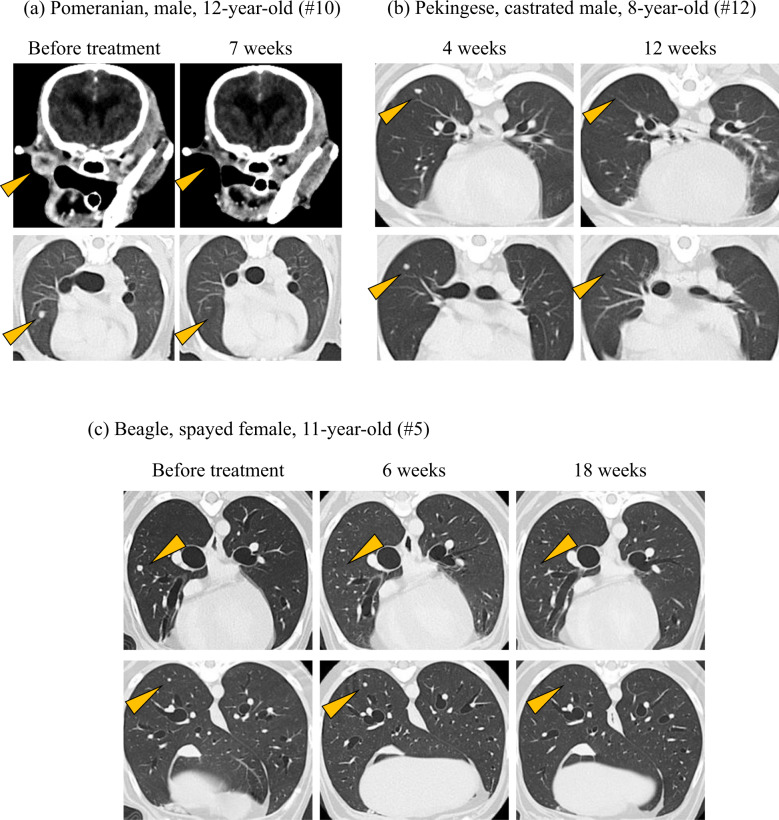
Antitumor efficacies of c4G12 in dogs with oral malignant melanoma. **a** Representative tumor response in dogs with measurable disease. The dog (Pomeranian, male, 12-year-old, #10 in Supplementary Tables 2, 3) received c4G12 at a dose of 5 mg/kg every 2 weeks. Oral recurrent tumor (upper panel) and pulmonary metastatic lesions (lower panel) responded to treatment at week 7. **b**, **c** Representative tumor response in dogs with non-measurable disease. **b** The dog (Pekingese, castrated male, 8-year-old, #12) received c4G12 at a dose of 5 mg/kg every 2 weeks. Pulmonary metastatic lesions were confirmed at week 4. Subsequently, the lesions responded to treatment at week 12. **c** The dog (Beagle, spayed female, 11-year-old, #5) received c4G12 at a dose of 5 mg/kg every 2 weeks until week 12 and thereafter the dose was changed to 2 mg/kg. A metastatic lesion in the lung (upper panel) disappeared at week 6. In contrast, the other lesion (lower panel) showed slight increase in tumor size at week 6, followed by complete regression at week 18 (an immune-related pattern of response). Contrast-enhanced and matched transverse CT images are shown. Arrow heads indicate tumor lesions.

As more than half of the dogs did not have measurable lesions at baseline, evaluation of tumor response was of secondary interest in this study. Tumor response as evidenced by diagnostic imaging was observed in 5 of 29 dogs (17.2%). According to cRECIST v1.0, one dog experienced a complete response (CR; dog #10) among 13 dogs that had measurable diseases at baseline, with ORR of 7.7% (95% confidence interval (CI) = 0.2–36.0%) (Table 4, Fig. 2a, and Supplementary Table 3). Other 4 dogs that experienced tumor response only had non-measurable lesions at baseline and thus the response could not be evaluated by cRECIST. However, all detectable tumors disappeared in 2 dogs (dog #12 and #19), leading to numerically long survival time of more than 1 year (417 days and >530 days, respectively; Fig. 2b and Supplementary Table 3). In the other 2 dogs (dog #5 and #28), all lung metastatic lesions disappeared in response to the treatment (Fig. 2c), but residual tumors persisted in the lymph nodes and/or oral cavity. Responses were durable, but all 5 dogs eventually had disease progression at later time-point. All deaths were considered tumor-related, except for dog #10 which died from chronic kidney disease at day 168 of c4G12 treatment.

**Table 4:** Evaluation of response to c4G12 treatment.

Best overall response—no. (%)	
CR	1 (7.7)
PR	0 (0)
SD	0 (0)
PD	10 (76.9)
NE	2 (15.4)
ORR―% (95% CI)	7.7 (0.2–36.0)

Tumor response to c4G12 treatment was defined and recorded according to cRECIST v1.0^30^.

*CR* complete response, *PR* partial response, *SD* stable disease, *PD* progressive disease, *NE* not evaluable, *ORR* objective response rate (CR + PR), *CI* confidence interval.

**Fig. 3:**
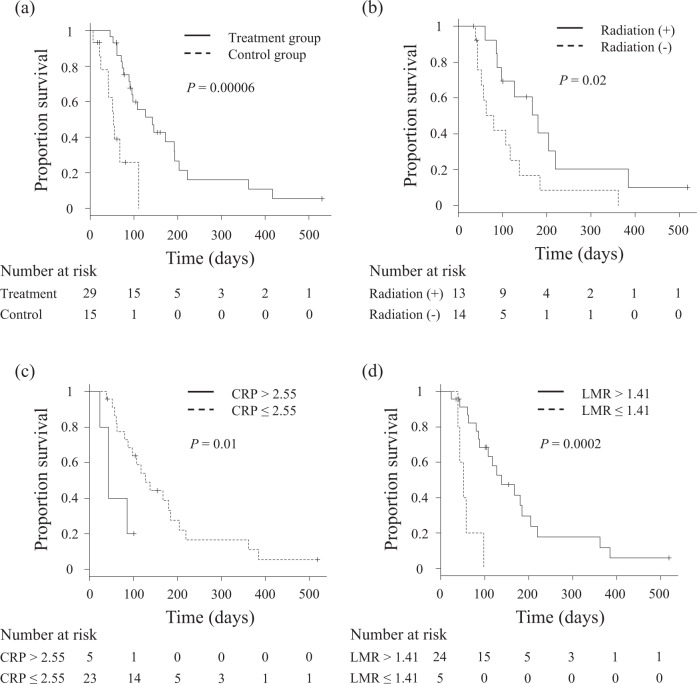
Survival of dogs treated with c4G12. **a** Comparison of survival between c4G12 treatment group (*n* = 29) and historical control group (*n* = 15). Survival (days) was defined as time from confirmation of pulmonary metastasis to death. For subgroup analysis, the treatment group was dichotomized according to (**b**) the use of previous/concomitant radiation, (**c**) plasma C reactive protein (CRP) levels (cutoff 2.55 mg/dL), or (**d**) the lymphocyte-to-monocyte ratio (LMR, cutoff 1.41), and overall survival was compared between the subgroups. Dogs that received radiation therapy concomitantly or ≤8 weeks before the initiation of antibody treatment were classified into radiation (+) subgroup. Marks on the line indicate censored data. Statistical analysis was performed using the log-rank test.

Survival from confirmation of PM to death was compared by the Kaplan–Meier method in the treatment group versus a historical control group (*n* = 15)^16^. Survival was significantly longer in the treatment group (*P* = 0.00006), with median survival of 143 days (95% CI of 91–194 days), in contrast to 54 days (95% CI of 25–NA days) in the control group (Fig. 3a; see Supplementary Table 4 for details of dogs in the historical control group). Subpopulation analysis only involving dogs with measurable disease confirmed a significant difference in survival between the treatment (*n* = 13) and historical control group (*n* = 8; *P* = 0.02). Similarly, in dogs with non-measurable disease, survival was longer in the treatment group (*P* = 0.01, Supplementary Fig. 4). In order to gain some insights into the factors that could predict survival, the treatment group was further dichotomized into subgroups according to the use of radiation therapy and peripheral blood immunological markers. The concomitant or previous (within 8 weeks of treatment initiation) use of radiation was significantly associated with improved overall survival (OS) in the c4G12 treatment group (*P* = 0.02; Fig. 3b). Moreover, OS with c4G12 treatment was significantly longer in dogs that had low plasma CRP (cutoff 2.55, *P* = 0.01) or high LMR (cutoff 1.41, *P* = 0.0002) at baseline (Fig. 3c, d).

## Discussion

Immunotherapy targeting the PD-1/PD-L1 axis is a promising treatment for several malignant cancers in humans. However, although a subset of patients has a remarkable response to treatment, around 60–80% of patients do not respond^1^. Exploration of resistance mechanisms and predictive biomarkers of immune checkpoint inhibitors (ICIs) is a growing field of study in human oncology. In dogs, only limited evidence for PD-L1 expression in cancers is available and the safety and efficacy of anti-PD-L1 therapy have not yet been robustly established. In the present report, we demonstrated PD-L1 expression in most types of canine malignant cancers with IHC. In dogs with pulmonary metastatic OMM, c4G12 treatment was safe and induced tumor response in some dogs. Importantly, the survival of dogs with c4G12 treatment was significantly longer than that of the historical control group. Because no systemic therapy that prolongs the survival of dogs with pulmonary metastatic OMM is currently available, the results of this study strongly encourage the further development of anti-PD-L1 therapy in dogs.

Except for transmissible venereal tumor, PD-L1 expression was detected in more than 70% of the samples, consistent with previous reports using different mAbs^19,20^. Moreover, the majority was classified as TPS ≥ 50%. These rates were high compared with human melanoma, squamous cell carcinoma, and urothelial cancer, in which 51–71% was PD-L1-positive and only 14–31% was TPS ≥ 50%^31^, suggesting the differences in etiology or immune microenvironment of cancers between dogs and humans. The reason PD-L1 expression was not detected in our samples of transmissible venereal tumor remains unclear. In another contagious cancer known as the Tasmanian devil facial tumor disease, tumor cells can express PD-L1 in vitro, but its expression is minimal in naturally infected animals^32^. As these unique cancers possess alternative mechanisms to evade the host immune system (e.g., downregulation of MHC molecules)^33–35^, immunosuppression via the PD-1/PD-L1 pathway might not be important, a topic to be further clarified. To date, PD-L1 expression in tumor biopsies is utilized to identify the eligible patient for anti-PD-1/PD-L1 therapy. For example, TPS ≥ 1% is required for NSCLC patients to receive pembrolizumab as first-line treatment. However, its reported predictive utility varies among studies^9–11,36,37^. In our study, the association between tumor PD-L1 expression and clinical outcome remains unclear, because most dogs had PD-L1-positive cancers and TPS ≥ 50%. Notably, among 5 dogs that responded to treatment, 1 had PD-L1 TPS of <1%, suggesting that PD-L1 IHC alone might not be an adequate biomarker. Larger studies are needed to confirm the predictive utility of PD-L1 IHC in canine anti-PD-L1 therapy.

The c4G12 treatment was well-tolerated, and the frequency and severity of TRAEs were consistent with previous reports of human clinical trials using anti-PD-1/PD-L1 mAbs^9,38^. TRAEs with potential immune-related causes included pneumonitis and thrombocytopenia. Hepatitis, pancreatitis, and colitis were suspected from other TRAEs in several dogs, although none of those was clinically confirmed. Since pneumonitis, colitis, hepatitis, endocrinopathies, and infusion-related reaction are reported in human anti-PD-1/PD-L1 therapies^9,38^, these events should be assessed in future canine studies. The ORR of 7.7% in c4G12 treatment was low compared with previous human studies of advanced melanoma using anti-PD-L1 mAbs, with 17.3%–30.2% patients having objected responses^38,39^. Tumor mutational burden (TMB) was reported to correlate with improved response to ICIs including anti-PD-L1 mAbs^40,41^. In human cutaneous melanoma, mean TMB was 49.17 mutations/Mb, while acral or mucosal melanoma had mean TMB of only 2.64 mutations/Mb^42^. Because canine oral melanoma arises independent of ultra-violet light exposure with TMBs comparable to human mucosal melanoma^43,44^, low response rate to c4G12 treatment might reflect low neoantigen expression in canine OMM. Further research is required to elucidate resistant mechanisms to anti-PD-L1 therapy in dogs.

The OS of dogs with c4G12 treatment was enhanced with the use of radiation therapy, as previously seen in mouse preclinical models^45^ and a human clinical study, where the previous radiotherapy was associated with the improved OS in NSCLC patients treated with pembrolizumab^22^. Because radiation induces immunogenic cell death in the tumor microenvironment (TME) and subsequently activates antitumor immune responses^46^, combined radiation and ICIs represent a promising strategy to improve treatment outcome, the topic of our future study on canine OMM using c4G12. Peripheral blood CRP level was also associated with improved OS in the c4G12 treatment group, consistent with a previous report of cancer patients who were treated with anti-PD-1/PD-L1 therapy^23^. In cancer patients, CRP levels can be increased^47^, possibly reflecting the inflammatory TME. Although inflammation can be tumor-promoting^48^, the reason animals with high CRP levels have poor survival remains unclear. Similarly, OS with c4G12 treatment was significantly longer in dogs with high LMR, as with a previous report on pembrolizumab treatment in metastatic melanoma patients^24^. LMR is a systemic immune status marker that, at least in part, reflects the TME^49,50^. Because tumor-infiltrating lymphocytes play a pivotal role in antitumor immunity and circulating monocytes can differentiate into tumor-associated macrophages that have protumor propensity^51^, increased LMR could be an indicator for preferable antitumor immune responses. Because these analyses were conducted in a univariate manner and were exploratory in nature with a limited sample size, further studies are needed to validate these findings.

The use of a small-sized historical control group in survival comparison is a limitation in this clinical study. Only dogs with pulmonary metastatic OMM which were recently treated in the same hospital were included in the control group; however, the results should be interpreted carefully because the control group was retrospective and non-randomized population that may contain multiple biases. Because prolongation of survival by c4G12 treatment was strongly suggested, further studies involving more appropriate control (e.g. double-blinded, randomized, and/or placebo-treated) are warranted to confirm the benefit in survival.

Although tumor-bearing mice are often utilized as preclinical models, results are not always reproduced in humans^52^. Because some canine naturally occurring cancers, including malignant melanoma, resemble human cancers^53^ and dogs are genetically outbred and immunocompetent, dogs might be useful large animal models, especially for immunological studies including ICIs.

In conclusion, PD-L1 appears to be a promising target for canine cancer immunotherapy, and c4G12, an immune checkpoint-inhibiting antibody for dogs, deserves further investigation. We believe that this study is a substantial step toward putting the immune checkpoint blockade into veterinary clinical practice and making dogs as a translational model for human cancer research.

## Methods

### Specimens and mAbs used for IHC

Formalin-fixed and paraffin-embedded (FFPE) tissue samples were used for PD-L1 IHC. The tissue samples had been submitted for histological diagnosis and stored in a commercial pathology laboratory (North Lab, Hokkaido, Japan). The study included samples of normal tonsil (*n* = 4), skin squamous cell carcinoma (*n* = 20), nasal adenocarcinoma (*n* = 20), transitional cell carcinoma (*n* = 20), anal sac gland carcinoma (*n* = 20), soft tissue sarcoma (*n* = 20), osteosarcoma (*n* = 20), OMM (*n* = 20), mammary adenocarcinoma (*n* = 20), histiocytic sarcoma (*n* = 20), diffuse large B-cell lymphoma (*n* = 20), gastric adenocarcinoma (*n* = 5) and transmissible venereal tumor (*n* = 4). Inflammatory mammary carcinoma samples were excluded.

A rat anti-PD-L1 mAb, 6G7-E1, has been described previously^19^. A new anti-PD-L1 mAb 6C11-3A11 was selected from previously established hybridoma clones^27^. Rat immunization, hybridoma establishment, isotyping, and mAb purification were performed at Cell Engineering Corporation (Osaka, Japan). The binding specificity of 6C11-3A11 was confirmed using recombinant canine PD-L1–expressing cells by flow cytometry in our previous paper^28^.

### PD-L1 IHC

FFPE sections underwent immunohistochemical staining with 6G7-E1 as previously described^19^, except for the use of non-decalcified specimens for osteosarcoma. Similarly, 6C11-3A11 staining was performed at a final concentration of 5 μg/mL. Histofine simple stain MAX PO (rat) (Nichirei, Tokyo, Japan) was used as a secondary antibody, and Mayer’s hematoxylin stain was used as a counterstain. When histological evidence of cell staining was observed by an optical microscope, the assessed sections were considered positive for PD-L1 expression. Stained sections without the primary antibody served as negative control for 6C11-3A11 IHC (Supplementary Fig. 5). Comparison of the anti-PD-L1 mAbs was performed by staining sections obtained from the same cancer samples. For 6C11-3A11 IHC, the PD-L1 TPS was defined and calculated as follows: TPS = the number of viable tumor cells with PD-L1 expression/the number of total viable tumor cells × 100 (%). The evaluation included a minimum of 100 viable tumor cells.

### Clinical study using c4G12

The present clinical study was conducted in the Veterinary Teaching Hospital, Hokkaido University as research aiming to demonstrate that PD-L1 is a promising target for cancer immunotherapy in dogs. The study was approved by the Institutional Animal Care Committee, Hokkaido University (Approval number: 15–0149) and the Faculty of Veterinary Medicine, Hokkaido University (Approval number: 15028). The use of animals throughout the clinical study was approved by the ethics committee, Faculty of Veterinary Medicine, Hokkaido University. Prior to study enrollment, written informed consent was obtained from both the dogs’ owner and veterinarian. Dogs were required to meet the following criteria for inclusion; (a) histologically confirmed malignant melanoma that originated in the oral cavity (b) with pulmonary metastatic lesion(s) as evidenced by chest X-ray or CT. Dogs with irrelevant severe systemic illness or autoimmune disease were excluded from the study. In total, 29 dogs were enrolled, including 4 dogs described in the previous report^16^. PD-L1 expression in the primary tumors, obtained by surgical excision at prior surgeries or biopsies, was assessed by 6C11-3A11 IHC. PD-L1 TPS was calculated as described above, as long as an adequate tumor section was available for evaluation. Dogs were treated with c4G12 every 2 weeks at 2 mg or 5 mg/kg by intravenous administration using a syringe pump over 1 h. The optimization of the dose and delivery regimen was not a scope of this study; the treatment dose was basically set as 5 mg/kg, but determined by veterinarians on a case-by-case basis. During the treatment, physical examination, complete blood count, and blood chemistry were performed at intervals of 2–6 weeks. When clinically necessary, concomitant drugs not considered to be immunosuppressive were allowed (e.g., NSAIDs, antibiotics, anodynes, and antitussives). In all dogs treated by concomitant or previous radiation therapy, the oral tumor and involved lymph nodes, if present, received approximately 26–32 Gy in total, which were delivered in 4 fractions at one-week intervals using Elekta Synergy integrity R LINAC (Elekta AB, Stockholm, Sweden) or TITAN-320S (Shimadzu Industrial System, Kyoto, Japan). The study period was from 11^th^ March 2016 to 11^th^ March 2020. Dogs still alive at the end of the study period were included in the survival analysis as censored data.

### Evaluation of adverse events

Adverse events, that emerged after initiation of therapy with a reasonable possibility that the event was caused by c4G12 treatment, were defined as treatment-related events. These events were graded and recorded following the veterinary cooperative oncology group–common terminology criteria for adverse events (VCOG-CTCAE) v1.1^54^.

### Evaluation of tumor response

Tumor response to the treatment was defined and recorded according to the response evaluation criteria in solid tumors in dogs (cRECIST) v1.0^30^. Assessment of the tumor burden was performed by X-ray or CT within 3 weeks prior to the first c4G12 dose (baseline) and was scheduled every 6 weeks during the treatment. The dogs that have no measurable (target) lesion at baseline were considered “with non-measurable disease” and excluded from the response evaluation. The ORR was calculated as the percentage of dogs that had the best overall response of CR and partial response (PR).

### Evaluation of survival

Survival duration (days) of dogs in the c4G12 treatment group was defined as time from confirmation of PM to death. Dogs with pulmonary metastatic OMM treated by standard therapies at the Veterinary Teaching Hospital of Hokkaido University during 2013 to 2016 were used as a historical control group^16^. All deaths in the control group were considered tumor-related. There was no statistically significant difference in sex, age, and body weight between the two groups (Fisher’s exact test for sex and Mann–Whitney U test for age/body weight). For subgroup analysis, OS (days) of the treated dogs was defined as time from the first dose of c4G12 to death. The dogs were divided into subgroups according to the use of previous (≤8 weeks from the first dose of c4G12) or concomitant radiation, and baseline plasma CRP and LMR. LMR was defined as the ratio of absolute lymphocyte count to absolute monocyte count. The baseline blood test was performed on the day of the first dose of c4G12. The optimal cutoff values for the CRP level and LMR was determined using receiver operating characteristic (ROC) curves (Supplementary Fig. 6). The survival curves were generated using the Kaplan–Meier method and statistical analysis was performed using the log-rank test. A statistical software EZR (version 1.35)^55^ was used in all analyses and *P* < 0.05 was considered statistically significant.

### Reporting summary

Further information on research design is available in the Nature Research Reporting Summary linked to this article.

## Supplementary information

supplementary materials

reporting summary

## Data Availability

High-resolution immunohistochemistry (IHC) and computerized tomography (CT) images supporting Figs. 1 and 2 of the article, and baseline blood test analysis data supporting Fig. 3, are available in the figshare repository: 10.6084/m9.figshare.13385441^56^. The datasets supporting the survival analysis plots in Fig. 3, and all other data supporting the findings of the current study, are included in the supplementary files that accompany the article.
