# Supplementary material for: PD-L1 immunohistochemistry for canine cancers and clinical benefit of anti-PD-L1 antibody in dogs with pulmonary metastatic oral malignant melanoma
Source: NPJ Precis Oncol. 2021 Feb 12;5:10. doi: 10.1038/s41698-021-00147-6 (PMC7881100; doi:10.1038/s41698-021-00147-6)
Supplement: Supplementary file 1 — supplementary materials [file 41698_2021_147_MOESM1_ESM.pdf]

PD-L1 immunohistochemistry for canine cancers and clinical benefit of anti-PD-L1 antibody in dogs with pulmonary metastatic oral malignant melanoma

Naoya Maekawa, Satoru Konnai*, Maki Nishimura, Yumiko Kagawa, Satoshi Takagi, Kenji Hosoya, Hiroshi Ohta, Sangho Kim, Tomohiro Okagawa, Yusuke Izumi, Tatsuya Deguchi, Yukinari Kato, Satoshi Yamamoto, Keiichi Yamamoto, Mikihiro Toda, Chie Nakajima, Yasuhiko Suzuki, Shiro Murata, Kazuhiko Ohashi

*Corresponding author
Satoru Konnai
E-mail: konnai@vetmed.hokudai.ac.jp

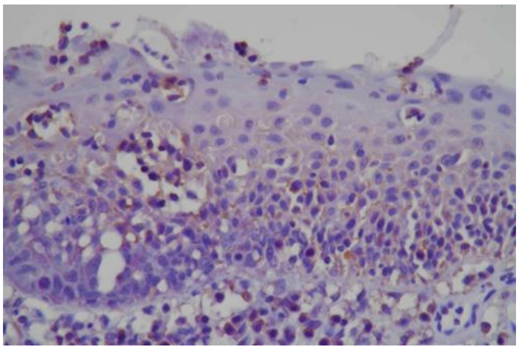

Supplementary Figure 1. 6C11-3A11 immunohistochemistry in canine tonsil. Sections were cut from formalin-fixed and paraffin-embedded tissues of normal tonsil (*n* = 4). Subsequently, sections were stained with 6C11-3A11. A representative IHC result is shown.

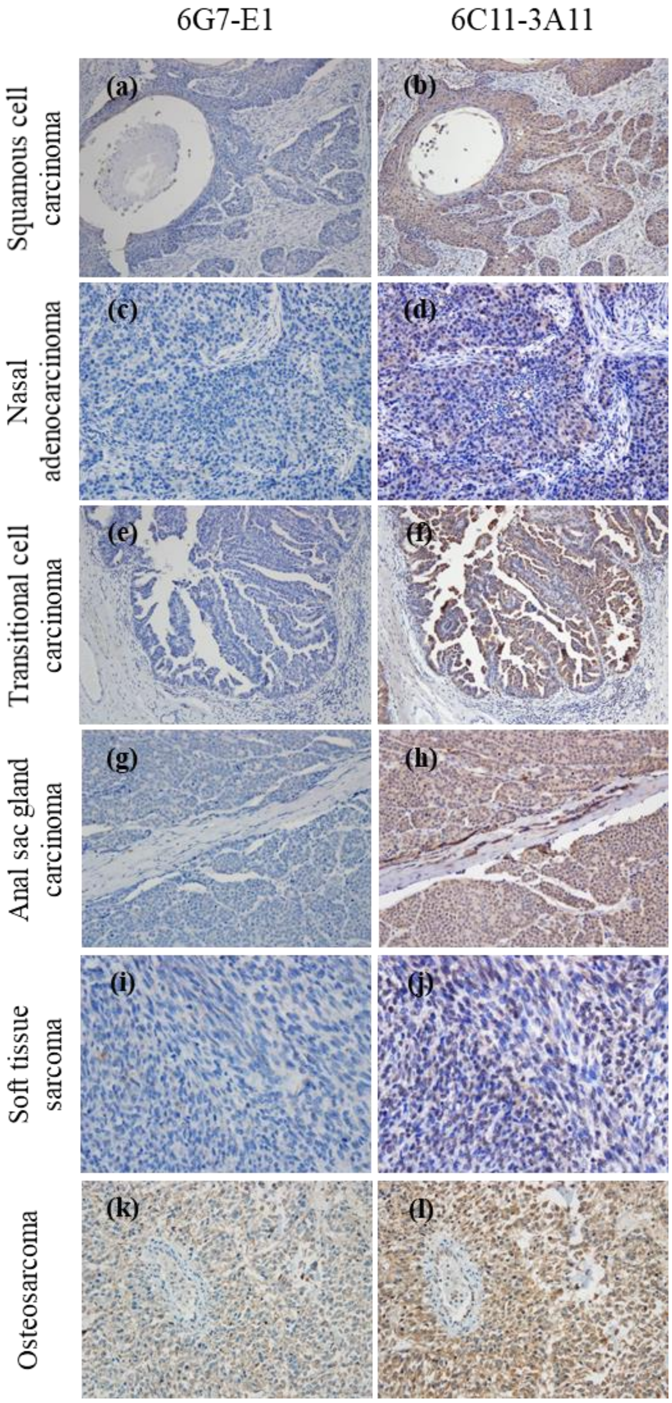

Supplementary Figure 2. Direct comparison of 6G7-E1 and 6C11-3A11 for PD-L1 immunohistochemistry. Sections were cut from identical formalin-fixed and paraffin-embedded cancer tissues. Subsequently, sections were stained with either 6G7-E1 (a, c, e, g, i, k) or 6C11-3A11 (b, d, f, h, j, l). Representative IHC results for each cancer type are shown. Original magnification, 100× (a, b, e, f), 200× (c, d, g, h, k, l), or 400× (i, j).

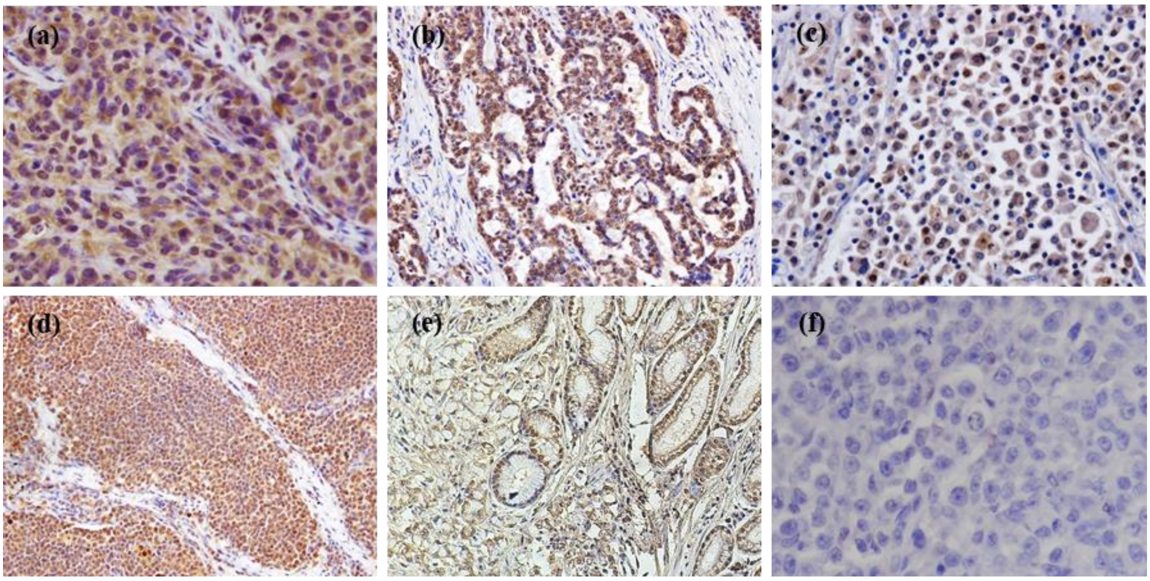

Supplementary Figure 3. PD-L1 immunohistochemistry with 6C11-3A11 in canine malignant cancers. Formalin-fixed and paraffin-embedded cancer tissues were stained with 6C11-3A11. Representative IHC results for (a) oral malignant melanoma, (b) mammary adenocarcinoma, (c) histiocytic sarcoma, (d) diffuse large B-cell lymphoma, (e) gastric adenocarcinoma, and (f) transmissible venereal tumor are shown. Original magnification, 100× (e), 200× (b, d), or 400× (a, c, f).

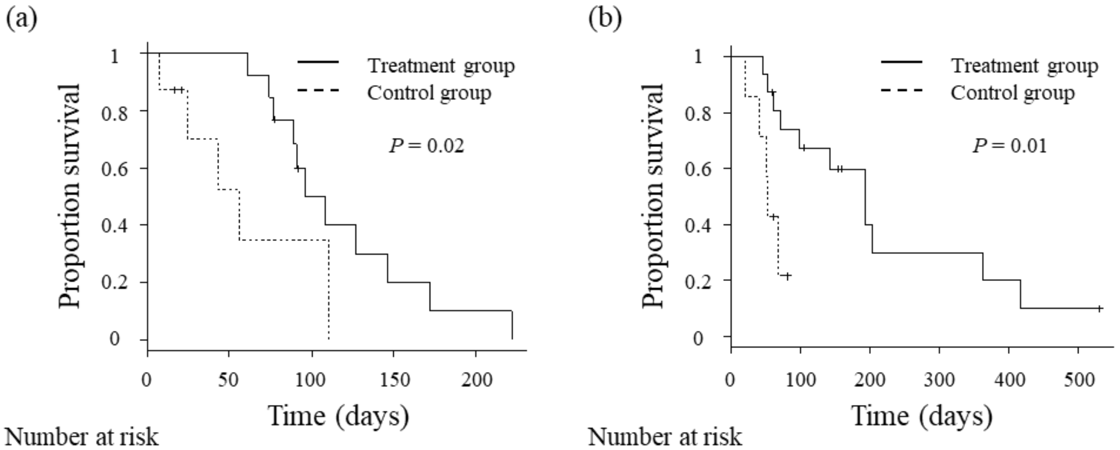

Number at risk (a)

Treatment	13	13	5	2	1
Control	8	3	1	0	0

Number at risk (b)

Treatment	16	10	4	3	2	1
Control	7	0	0	0	0	0

Supplementary Figure 4. Survival analysis in dogs with measurable or non-measurable disease. Thirteen dogs in the treatment group and 8 dogs in the historical control group had measurable diseases at baseline assessment. (a) Comparison of survival in dogs with measurable disease between c4G12 treatment (*n* = 13) and historical control (*n* = 8) group. (b) Comparison of survival in dogs with non-measurable disease between c4G12 treatment (*n* = 16) and historical control group (*n* = 7). Survival (days) was defined as time from confirmation of pulmonary metastasis to death. Marks on the line indicate censored data. Statistical analysis was performed using the log-rank test.

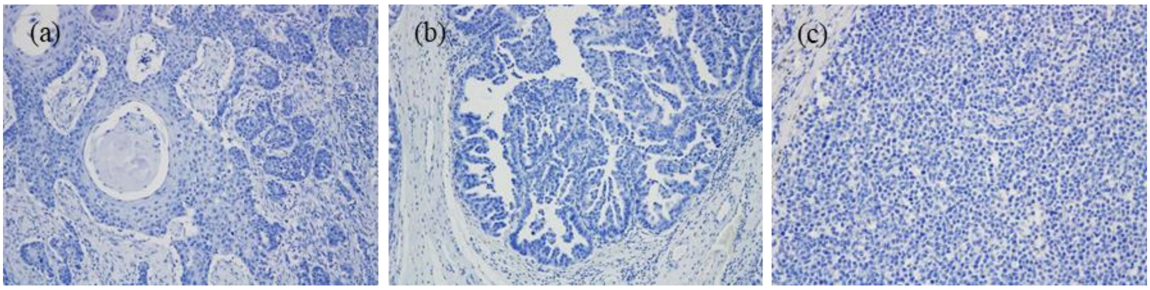

Supplementary Figure 5. Representative immunohistochemistry results without the primary antibody. Sections of (a) squamous cell carcinoma, (b) transitional cell carcinoma, and (c) diffuse large B-cell lymphoma were stained without the primary antibody as negative control stain. Original magnification, 100× (a, b) or 200× (c).

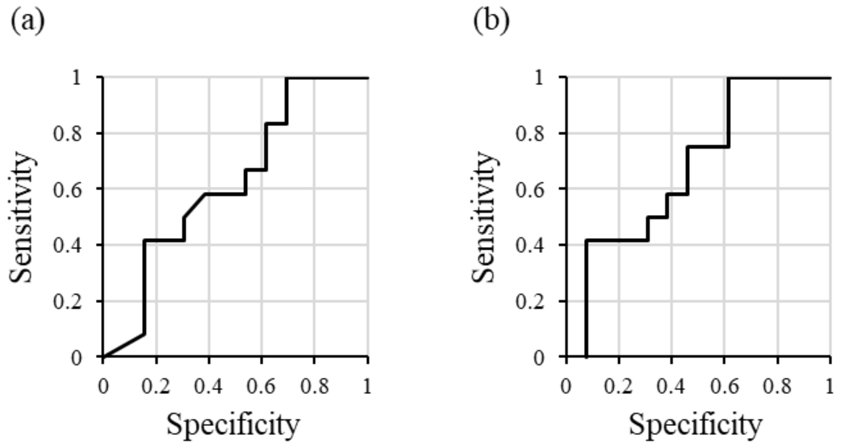

Supplementary Figure 6. Determination of optimal cut-off values using receiver operating characteristic curves. Youden's index was calculated to estimate the optimal cut-off. (a) ROC curve for CRP (AUC = 0.63, optimal cut-off = 2.55 mg/gL). (b) ROC curve for LMR (AUC = 0.68, optimal cut-off = 1.41).

Supplementary Table 1. Comparison of two anti-PD-L1 mAbs in canine cancer immunohistochemistry.

Pathology	6G7-E1		6C11-3A11	
	Positive no./Tested no.	Positive rate (%)	Positive no./Tested no.	Positive rate (%)
Squamous cell carcinoma (skin)	0/5	0	5/5	100
Nasal adenocarcinoma	0/5	0	5/5	100
Transitional cell carcinoma	0/5	0	5/5	100
Anal sac gland carcinoma	0/5	0	5/5	100
Soft tissue sarcoma	0/5	0	4/5	80
Osteosarcoma	5/5	100	5/5	100

Supplementary Table 2. Characteristics of dogs enrolled in the clinical study.

Dog #	Breed	Sex	Age (year)	Tumor PD-L1	PD-L1 TPS	Prior therapy	Concurrent disease	Remark
1	Pug	Male, castrated	11	+	≥50%	Surgery	None	Dog no. 2 in ref.(16)
2	Miniature dachshund	Male, castrated	14	+	ND	Radiation	None	Dog no. 4 in ref.(16)
3	Golden retriever	Male, castrated	10	+	ND	Surgery	None	Dog no. 5 in ref.(16)
4	Miniature dachshund	Male, castrated	14	+	ND	Radiation	None	Dog no. 6 in ref.(16)
5	Beagle	Female, spayed	11	+	≥50%	Surgery	None	
6	American cocker spaniel	Male, castrated	13	+	≥50%	Surgery	Chronic kidney disease, hypothyroidism, chronic pancreatitis	
7	Yorkshire terrier	Male	14	+	≥50%	Surgery	Inguinal hernia, intervertebral disk herniation	
8	Kaninchen dachshund	Female, spayed	13	+	ND	Surgery, chemotherapy	None	
9	Miniature dachshund	Male	13	+	≥50%	Surgery	Hypothyroidism, megaesophagus, atopic dermatitis	

10	Pomeranian	Male	12	+	1-49%	Surgery, radiation	Polycystic kidney, dilated cardiomyopathy	
11	Mix	Female	12	-	<1%	Surgery	None	
12	Pekingese	Male, castrated	8	+	≥50%	Radiation	Keratoconjunctivitis sicca	
13	Mix	Male, castrated	11	+	≥50%	Radiation	None	
14	Miniature dachshund	Male	13	+	≥50%	Surgery, radiation	None	
15	Miniature dachshund	Female	15	+	≥50%	Surgery	Pyometra	
16	Labrador retriever	Female	11	+	≥50%	Radiation	None	
17	American cocker spaniel	Male	10	+	≥50%	Radiation	None	
18	Toy poodle	Female, spayed	13	+	≥50%	Radiation	Myxomatous mitral valve degeneration	
19	Miniature dachshund	Female, spayed	15	-	<1%	Surgery, radiation	None	
20	Miniature dachshund	Male	14	ND	ND	Radiation	None	

21	Labrador retriever	Male	12	+	≥50%	Surgery, radiation	None	
22	Toy poodle	Male	16	+	≥50%	Radiation	None	
23	Chihuahua	Female, spayed	13	+	≥50%	Surgery	Myxomatous mitral valve degeneration	
24	Pug	Female, spayed	14	+	≥50%	Surgery	None	
25	Welsh Corgi	Male, castrated	10	+	≥50%	None	Pituitary macroadenoma, Cushing's disease	
26	Papillon	Male, castrated	14	+	≥50%	Surgery	None	
27	Flat-coated retriever	Female, spayed	8	+	ND	Surgery	None	
28	Golden retriever	Male, castrated	14	+	ND	Radiation	None	
29	Miniature dachshund	Male, castrated	14	+	ND	None	hydronephrosis	

TPS, tumor proportion score; ND, not determined.

Prior therapy included: definitive/palliative surgery; definitive/palliative radiation; chemotherapy with metronomic cyclophosphamide.

Supplementary Table 3. Summary of c4G12 treatment.

Dog #	Number of c4G12 doses	Treatment duration (day)	Amount of dosage	Concomitant therapy	Survival duration* (day)	Measurable lesion	Best overall response
1	16	220	Weeks 0–24: 2 mg/kg, then 5 mg/kg	Radiation	222	Present	PD
2	3	42	2 mg/kg	None	96	Present	PD
3	5	72	2 mg/kg	Radiation	91	Present	PD
4	6	82	5 mg/kg	None	89	Present	PD
5	25	335	Weeks 0–12: 5 mg/kg, then 2 mg/kg	Lymphadenectomy	362	Absent	-**
6	7	98	5 mg/kg	None	108	Present	PD
7	4	44	5 mg/kg	None	46	Absent	-
8	2	35	5 mg/kg	None	92†[a]	Present	NE
9	3	42	5 mg/kg	(Radiation, chemotherapy) ‡	146	Present	PD
10	11	168	5 mg/kg	None	172	Present	CR
11	12	180	5 mg/kg	Radiation	194	Absent	-
12	26	385	5 mg/kg	Radiation	417	Absent	-**
13	5	61	5 mg/kg	None	61	Absent	-
14	13	204	5 mg/kg	Lymphadenectomy, surgery, radiation	204	Absent	-
15	2	28	5 mg/kg	None	71	Absent	-

16	6	98	2 mg/kg	None	98	Absent	-
17	7	101	5 mg/kg	None	160†[a]	Absent	-
18	3	35	5 mg/kg	None	74	Present	PD
19	38	518	5 mg/kg	Surgery	530†[b]	Absent	-**
20	8	118	5 mg/kg	None	127	Present	PD
21	2	24	2 mg/kg	None	78†[a]	Present	NE
22	7	105	5 mg/kg	None	105†[a]	Absent	-
23	13	185	5 mg/kg	None	192	Absent	-
24	2	26	5 mg/kg	None	53	Absent	-
25	5	63	5 mg/kg	None	77	Present	PD
26	1	15	5 mg/kg	None	60†[a]	Absent	-
27	10	138	2 mg/kg	None	143	Absent	-
28	12	154	2 mg/kg	None	154†[b]	Absent	-**
29	3	42	5 mg/kg	None	61	Present	PD

PD, progressive disease; CR, complete response; NE, not evaluable.

*Survival duration was defined as time from confirmation of pulmonary metastasis (PM) to death.

**At least one non-measurable lesions disappeared in response to the treatment.

†Censored data due to (a) loss to follow-up/withdrawal of consent or (b) the end of study period.

‡Treatment with c4G12 was halted on day 42 due to the disease progression. The dog was subsequently treated with radiation, followed by metronomic cyclophosphamide.

Supplementary Table 4. Characteristics of dogs in the historical control group.

Dog #	Breed	Sex	Age (year)	Survival duration* (day)	Therapy before PM	Therapy after PM
1	Miniature dachshund	Female	unknown	41	Surgery, chemotherapy, radiation therapy	None
2	Chihuahua	Female	13	7	Surgery	None
3	Golden retriever	Female, spayed	14	17†	None	Surgery, radiation therapy
4	Beagle	Male, castrated	14	21†	None	Surgery
5	Pomeranian	Male	13	51	Surgery, radiation therapy	None
6	Mix	Male	10	56†	Surgery, chemotherapy	Surgery, chemotherapy
7	Miniature dachshund	Male, castrated	14	68	Surgery, chemotherapy	Chemotherapy, radiation therapy
8	Miniature schnauzer	Male	10	22	Surgery, chemotherapy	Chemotherapy
9	Miniature dachshund	Female, spayed	13	25	Surgery	Radiation therapy
10	Miniature dachshund	Female, spayed	14	56	Surgery	Radiation therapy
11	Miniature dachshund	Male	12	43	Surgery, chemotherapy	None

12	Miniature dachshund	Female, spayed	16	81†	Radiation therapy	None
13	Miniature dachshund	Female, spayed	14	62†	Surgery	Chemotherapy
14	Miniature dachshund	Male, castrated	14	54	Surgery, radiation therapy	Chemotherapy
15	Miniature dachshund	Male	14	111	None	Surgery

Dogs were treated with definitive/palliative surgery, definitive/palliative radiation therapy and/or chemotherapy using cyclophosphamide/carboplatin at the same veterinary hospital during 2013–2016.

*Survival duration was defined as time from confirmation of PM to death.

†Censored data.
